# The Adrenal Medulla Modulates Mechanical Allodynia in a Rat Model of Neuropathic Pain

**DOI:** 10.3390/ijms21218325

**Published:** 2020-11-06

**Authors:** Marina Arribas-Blázquez, Luis Alcides Olivos-Oré, María Victoria Barahona, Aneta Wojnicz, Ricardo De Pascual, Mercedes Sánchez de la Muela, Antonio G. García, Antonio R. Artalejo

**Affiliations:** 1 Department of Pharmacology and Toxicology, Veterinary Faculty and Instituto Universitario de Investigación en Neuroquímica, Universidad Complutense de Madrid, 28040 Madrid, Spain; marina.arribas@vet.ucm.es (M.A.-B.); olivos@ucm.es (L.A.O.-O.); vbg@ucm.es (M.V.B.); 2 Instituto de Investigación Sanitaria San Carlos, 28040 Madrid, Spain; 3 Departamento de Farmacología, Instituto Teófilo Hernando, Facultad de Medicina, Universidad Autónoma de Madrid, 28029 Madrid, Spain; aneta.wojna@gmail.com (A.W.); ricardo.pascual@uam.es (R.D.P.); agg@uam.es (A.G.G.); 4 Instituto de Investigación Sanitaria, Hospital Universitario de La Princesa, 28006 Madrid, Spain; 5 Department of Animal Medicine and Surgery, Veterinary Faculty, Universidad Complutense de Madrid, 20040 Madrid, Spain; sdlmuela@vet.ucm.es

**Keywords:** neuropathic pain, chromaffin cells, adrenal medulla, PNMT, stress

## Abstract

We have investigated whether the stress response mediated by the adrenal medulla in rats subjected to chronic constriction injury of the sciatic nerve (CCI) modulates their nocifensive behavior. Treatment with SK29661 (300 mg/kg; intraperitoneal (I.P.)), a selective inhibitor of phenylethanolamine N-methyltransferase (PNMT) that converts noradrenaline (NA) into adrenaline (A), fully reverted mechanical allodynia in the injured hind paw without affecting mechanical sensitivity in the contralateral paw. The effect was fast and reversible and was associated with a decrease in the A to NA ratio (A/NA) in the adrenal gland and circulating blood, an A/NA that was elevated by CCI. 1,2,3,4-tetrahydroisoquinoline-7-sulfonamide (SKF29661) did not affect exocytosis evoked by Ca^2+^ entry as well as major ionic conductances (voltage-gated Na^+^, Ca^2+^, and K^+^ channels, nicotinic acetylcholine receptors) involved in stimulus-secretion coupling in chromaffin cells, suggesting that it acted by changing the relative content of the two adrenal catecholamines. Denervation of the adrenal medulla by surgical splanchnectomy attenuated mechanical allodynia in neuropathic animals, hence confirming the involvement of the adrenal medulla in the pathophysiology of the CCI model. Inhibition of PNMT appears to be an effective and probably safe way to modulate adrenal medulla activity and, in turn, to alleviate pain secondary to the injury of a peripheral nerve.

## 1. Introduction

Chromaffin cells from the adrenal medulla, the amplifying arm of the sympathetic nervous system (SNS), participate in stress responses by releasing the content of their secretory granules (mainly adrenaline (A), noradrenaline (NA), ATP, opioids, and chromogranins) into the bloodstream [1,2]. Control of chromaffin cell secretion is exerted by transmitters (acetylcholine (ACh), pituitary adenylate cyclase-activating peptide, ATP) released from synaptic terminals of the splanchnic nerve as well as by paracrine (their own secretion products) and endocrine substances (i.e., corticosteroids, histamine) [3]. Stimulus-secretion coupling in chromaffin cells begins when ACh released from splanchnic nerve terminals binds to nicotinic acetylcholine receptors (nAChRs) located at the chromaffin cell plasma membrane. This leads to the opening of the cation channel associated with nAChRs with the ensued generation of a postsynaptic excitatory potential, which eventually triggers the discharge of an action potential. Action potential depolarization depends on the activation of voltage-gated Na^+^ (Na_v_) and Ca^2+^ (Ca_v_) channels, whereas action potential repolarization relies on the activation of different classes of potassium channels (voltage- and/or calcium-gated K^+^ (K_v_, SK, and BK) channels. Importantly, Ca^2+^ entry during action potentials is essential to stimulate the exocytotic release of A and NA from chromaffin cells [4].

Interestingly, the adrenal medulla undergoes morphological and functional remodeling in rats subjected to chronic stress induced by cold (5 days at 4 °C) and chronic constriction injury of the sciatic nerve (CCI), a well-established model of neuropathic pain. In these conditions, the adrenal medulla exhibits an increased density of cholinergic nerve terminals and augmented frequency of spontaneous excitatory postsynaptic currents (sEPSCs), as well as enhanced exocytosis evoked by Ca^2+^ entry. In addition, exogenous application of ACh to chromaffin cells gives rise to enlarged nAChRs-mediated currents due to an increased expression of nAChRs [5,6]. Adrenomedullary chromaffin cells from CCI animals also display other forms of plasticity, such as an increased functional expression of P2X3 receptors and TRPV1 channels, which are reminiscent of those observed in neurons of L5 dorsal root ganglion from these same animals [7,8].

On the other hand, it is well known that stress may affect pain sensation. So, intense acute stress reduces pain perception and suppresses pain behavior [9,10,11]. At variance, certain forms of chronic stress (immobilization, cold, etc.) exacerbate (hyperalgesia) or even generate (allodynia) pain [12,13]. Both the hypothalamus–pituitary–adrenal cortex and the SNS are involved in acute and chronic stress responses, and their influence in pain states has been well documented [14,15]. Pain that depends on the activity of the SNS is called “sympathetically maintained pain” (SMP) and includes spontaneous pain and pain evoked by mechanical or thermal stimuli. It may be present in patients with the complex regional syndrome that follows the injury of an arm or leg [16]. Causal involvement of SNS is inferred from the pain-relieving effect of blocking transmission in sympathetic paravertebral ganglia and the ability A or NA injection to rekindle pain that has been relieved by sympathetic block. These findings are interpreted as that primary afferent nociceptive neurons are excited or possibly sensitized by A and/or NA [17]. Coupling of SNS to nociceptive neurons can occur in the periphery, in the axon, and in the dorsal root ganglion [18,19], and involve rather different mechanisms. So, sympathetic postganglionic terminals could mediate sensitization of nociceptive afferents to mechanical stimulation subsequently to nerve lesion and during inflammation. Accordingly, sensitization may depend on the activity of sympathetic neurons and on functional adrenoceptors expressed by afferent neurons, but also on inflammatory mediators, such as bradykinin, tumor necrosis factor, and interleukins 1, 6, and 8, which induce the release of prostaglandin E2 from sympathetic varicosities that, in turn, acts on nociceptors [20,21]. As a reflection of this, pharmacological strategies to alleviate pain secondary to injury of peripheral nerves focus not only on the reduction in SNS activity but increasingly on the modulation of the inflammatory process [22,23]. However, the contribution of the adrenal medulla to SMP has attracted much less attention [24]. Persistent activation of the adrenal medulla by chronic unpredictable sound stress on subdiaphragmatic vagotomy has been reported to generate mechanical hyperalgesia and enhance bradykinin-induced mechanical hyperalgesia in rats. Interestingly, denervation of the adrenal medulla after vagotomy decreases mechanical hyperalgesia [25,26]. Likewise, the application of a 2-adrenoceptor blocker prevents the enhancement of hyperalgesic behavior generated by the activation of the adrenal medulla, thereby implying this adrenoceptor in nociceptor sensitization.

Here, using a selective inhibitor of phenylethanolamine N-methyltransferase (PNMT), the enzyme that converts NA into A, and surgical denervation of the adrenal medulla, we have addressed the role of the adrenal medulla in neuropathic pain induced by CCI in the rat. Our results support the idea that chromaffin cell activity modulates nocifensive behavior in this experimental model and opens new opportunities for a pathophysiology-based treatment of neuropathic pain induced by the lesion of peripheral nerves.

## 2. Results

### 2.1. Effect of SKF29661 on Mechanical Allodynia 

As expected from previous results, mechanical allodynia was observed only in the CCI-injured hind paw, hence allowing the use of non-operated animals and the uninjured hind paw of CCI animals as controls (data not shown [8]). Importantly, the decrease in the paw withdrawal threshold (PWT) to tactile stimulation was fairly stable from day 7 to day 21 post-CCI surgery. Therefore, the drug’s effects in vitro and in vivo were studied during this time period.

**Figure 1:**
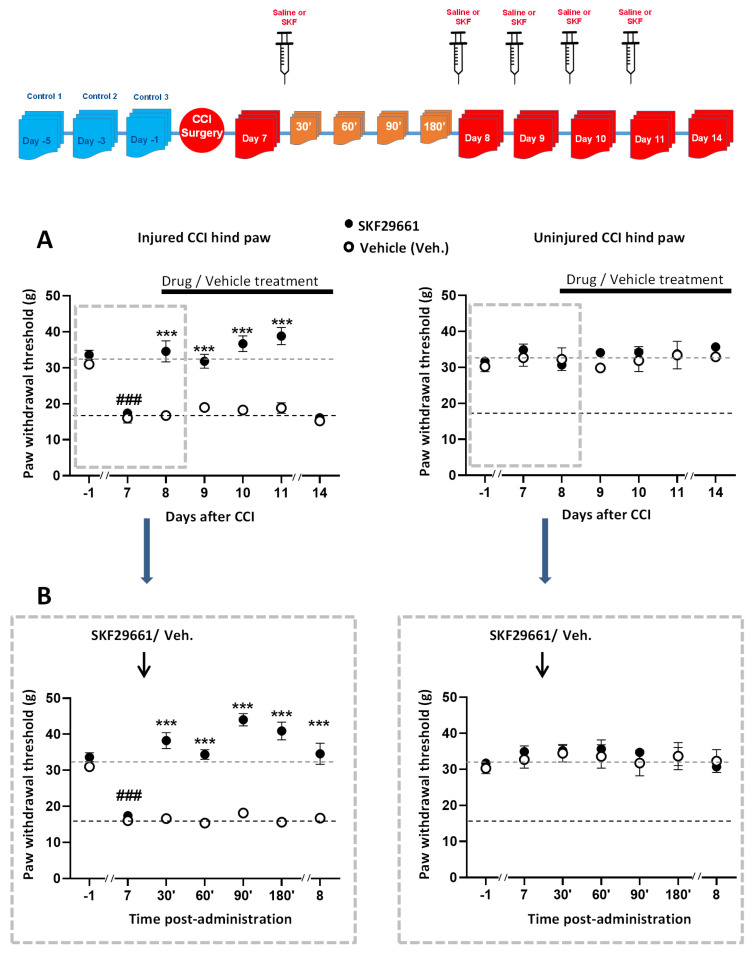
Effect of SKF29661 on mechanical allodynia from chronic constriction injury (CCI) animals. A graphical scheme of the experimental design is depicted on top of the figure. SKF29661 (300 mg/kg in 250 µL) or vehicle (saline) were injected intraperitoneal (I.P.) every 24 h (approximately at noon) for four consecutive days to CCI animals starting at day 7 after CCI surgery. Pre-CCI values (designated as −1) were taken as the mean of three consecutive determinations performed on days −5, −3, and −1 with regard to CCI surgery. Pharmacological testing was conducted on days 7–14 after surgery. Behavioral evaluation was generally performed 24 h after drug/vehicle administration (**A**) and also at 30 min, 60 min, 90 min, and 180 min following the first administration of SKF29661 (**B**). Data are expressed as the mean ± SEM of 6 (vehicle) or 12 (SKF29661) animals. Statistical significance was assessed by two-way ANOVA for repeated measures followed by a Tukey post hoc test for comparisons at matched times (***: *p* < 0.001). The statistical significance of the effect of CCI (7 days after CCI) with respect to pre-CCI values (−1) was evaluated by a one-way ANOVA for repeated measures followed by a Tukey post hoc test. (^###^: *p* < 0.001 for 7 days after CCI with respect to pre-CCI values (−1)).

1,2,3,4-tetrahydroisoquinoline-7-sulfonamide (SKF29661) is a selective and competitive inhibitor of PNMT that does not cross the blood–brain barrier [27,28]. To evaluate the involvement of the adrenal medulla in tactile hypersensitivity in CCI animals, we administered 300 mg/kg SKF29661 (*n* = 12 rats) or saline (*n* = 6 rats) intraperitoneal (I.P.) [27,28]. SKF29661 exerted a mechanical antiallodynic effect in the CCI-operated hind paw, which raised the PWT to pre-CCI levels (Figure 1A). The effect could be reproduced upon repetitive administration of SKF29661 at 24 h intervals. Interestingly, the onset of the effect of SKF29661 was surprisingly fast, starting 30 min after injection and lasting for the following 24 h (Figure 1B).

Likewise, the antiallodynic effect of SKF29661 was reversible, disappearing within the following 96 h after administration (see response at day 14 post-CCI). On the other hand, SKF29661 did not affect the nocifensive responses in the uninjured paw, suggesting it lacks an antinociceptive effect. As expected, vehicle injection did not modify behavioral responses in neither the CCI-injured hind paw nor the uninjured one.

**Figure 2:**
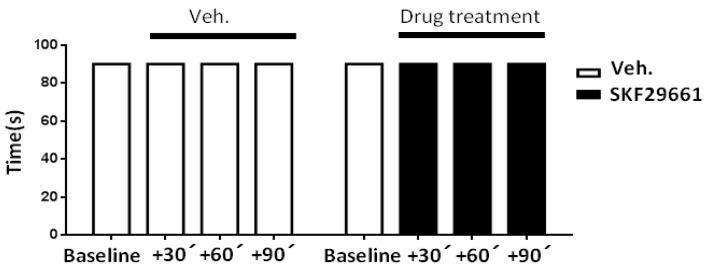
Effect of SKF29661 on motor coordination from Control animals. The graph depicts the time to fall from the RotaRod cylinder before (Baseline) and at 30, 60, and 90 min after administration of SKF29661 (300 mg/kg, I.P.) or vehicle (saline; Veh.) in three Control, non-operated, rats.

Although the results obtained in the uninjured hind paw of CCI animals suggested that SKF29661 does not affect motor coordination, this issue was directly investigated with the RotaRod test. In Control, non-operated animals, SKF29661 (300 mg/kg) did not affect the time to fall of the animals evaluated at 30, 60, and 90 min after I.P. administration (Figure 2). This result was reproduced along the 4 days of SKF29661 treatment (data not shown), thereby excluding an effect of the PNMT inhibitor on motor-coordination that could influence nocifensive responses.

**Figure 3:**
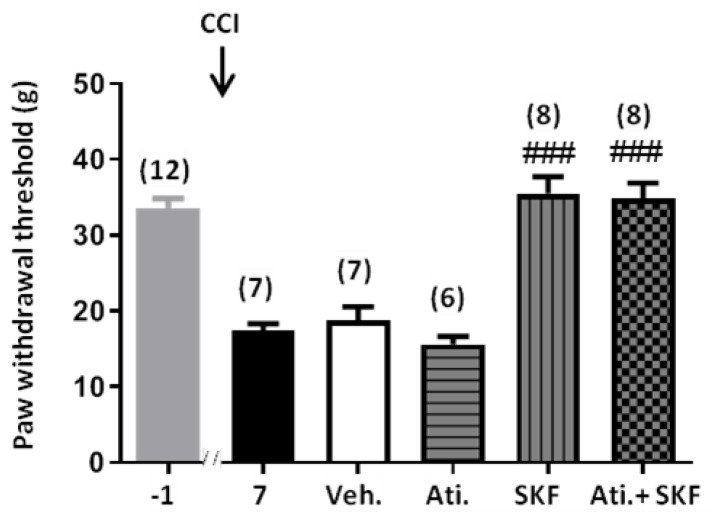
Contribution of α2 adrenergic receptors to mechanical allodynia from CCI animals. The effect of atipamezole (1 mg/kg, I.P.; Ati.) and vehicle (saline; Veh.) on mechanical allodynia was evaluated in the CCI-injured hind paw 30 min after their administration to both naive and SKF29661-treated (300 mg/kg, I.P.; SKF) animals. Pre-CCI (−1) values were taken as the mean of three consecutive determinations performed on days −5, −3, and −1 with regard to CCI surgery. Pharmacological evaluation was conducted on days 7–21 after surgery. Data are expressed as the mean ± SEM of the number of animals, which is shown between parentheses for each condition. Statistical significances of the drug’s effects were assessed with respect to vehicle by using a Student’s *t*-test for independent samples (^###^: *p* < 0.001).

Considering the ability of some PNMT inhibitors to interact with α2 adrenergic receptors [29] and the well-known antinociceptive and sedative effect of α2 adrenergic receptor agonists (e.g., dexmedetomidine, medetomidine, xylazine) [30], the effect of SKF29661 on mechanical allodynia was evaluated in rats treated with atipamezole (1 mg/kg, I.P.), an antagonist of α2 adrenergic receptors [30]. Atipamezole, neither on its own nor in the presence of SKF29661, modified the PWT to mechanical stimulation in the CCI-injured hind paw, hence excluding a significant contribution of α2 adrenergic receptors to both neuropathic pain and the antiallodynic effect of SKF29661 (Figure 3). Likewise, atipamezole, SKF29961, and their combination lacked an effect on mechanical sensitivity in the non-injured hind paw of CCI animals (data not shown).

### 2.2. Effect of SKF29661 on Adrenal and Blood Catecholamines

**Figure 4:**
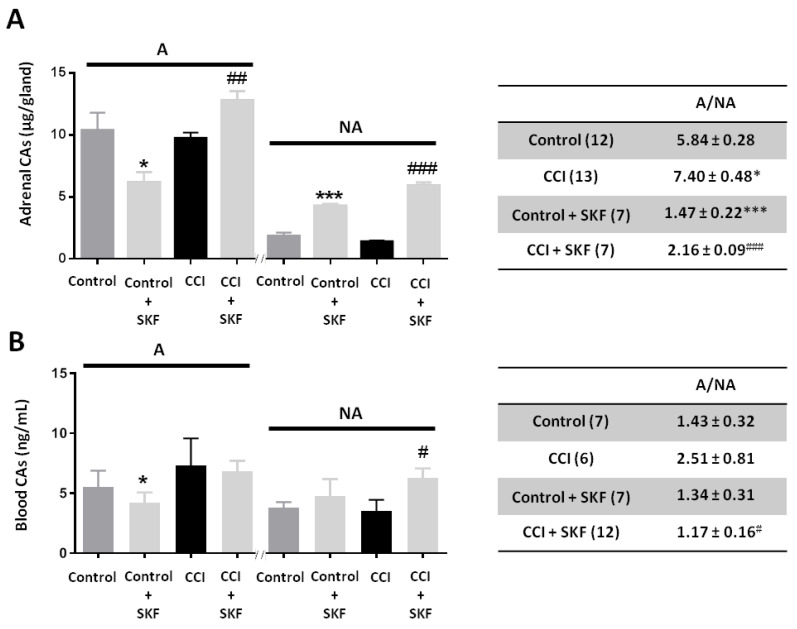
Effect of SKF29661 on adrenal and blood catecholamines (CAs) from Control and CCI animals. Animals were sacrificed between day 7 (CCI) and day 11 after CCI (at the end of treatment with SKF29661). Bar graphs represent A and NA content of the adrenal gland (**A**) or blood A or NA levels (**B**) for each condition. The number of glands (1 gland per animal) or blood samples and the A/NA for each condition is shown between parentheses in the accompanying tables. Data are expressed as mean ± SEM. Statistical significances with respect to Control (*: *p* < 0.05; ***: *p* < 0.001) or to CCI (^#^: *p* < 0.05; ^##^: *p* < 0.01; ^###^: *p* < 0.001) were assessed by a Student’s *t*-test for independent samples. Non-significant differences (*p* > 0.05) are not shown. A: Adrenaline; NA: Noradrenaline; SKF: SKF29661.

The adrenal content and blood levels of catecholamines (CAs) from Control (unoperated) and CCI animals treated and untreated with SKF29661 were determined. CCI reduced the adrenal gland content of both A (9.75 ± 0.44 μg/gland; *n* = 13 rats) and NA (1.39 ± 0.10 μg/gland; *n* = 13 rats) when compared to Control animals (10.39 ± 1.43 μg/gland and 1.84 ± 0.29 μg/gland for A and NA, respectively; *n* = 12 rats). It is worth noting that changes in A and NA content were not in parallel, as manifested by the A to NA ratio (A/NA). So, this ratio was 5.84 ± 0.28 in Control animals, whereas it rose to 7.40 ± 0.48 in CCI animals (Figure 4A). SKF29661 treatment (300 mg/kg, I.P., every 24 h for 4 days) significantly reduced A/NA in both Control (1.47 ± 0.22; *n* = 7 rats) and CCI (2.16 ± 0.09; *n* = 7 rats) animals, as expected from PNMT inhibition [27,31]. In Control rats, the change in A/NA was related to a reduction in the A content from 10.39 ± 1.42 μg/gland to 6.17 ± 0.84 μg/gland and to an increase in the content of NA from 1.84 ± 0.29 μg/gland up to 4.26 ± 0.18 μg/gland. In CCI animals, SKF29661 increased the content of A from 9.75 ± 0.44 μg/gland to 12.78 ± 0.75 μg/gland and the NA content from 1.39 ± 0.10 μg/gland to 5.91 ± 0.26 μg/gland (Figure 4A).

Blood A levels in CCI animals (7.33 ± 2.24 ng/mL; *n* = 6 rats) were higher than in Control ones (5.48 ± 1.40 ng/mL; *n* = 7 rats), whereas NA levels were slightly reduced (3.53 ± 0.92 ng/mL) as compared to Control animals (3.78 ± 0.47 ng/mL). Accordingly, the A/NA increased in CCI animals (2.51 ± 0.81) with respect to Control ones (1.43 ± 0.32) (Figure 4B). SKF29661 also affected blood CAs. In Control animals, SKF29661 treatment was associated to a significant reduction in circulating A (4.21 ± 0.84 ng/mL; *n* = 7 rats) and to an increase in NA (4.76 ± 1.42 ng/mL; *n* = 7 rats). In CCI animals, SKF29661 slightly reduced blood A (6.79 ± 0.91 ng/mL; 12 rats) but markedly increased NA (6.29 ± 0.77 ng/mL; *n* = 12 rats). Consequently, SKF29661 decreased the A/NA both in Control (1.34 ± 0.31) and in CCI (1.17 ± 0.16) animals (Figure 4B).

### 2.3. Effect of SKF29661 on Ionic Conductances and Exocytosis from Chromaffin Cells

**Figure 5:**
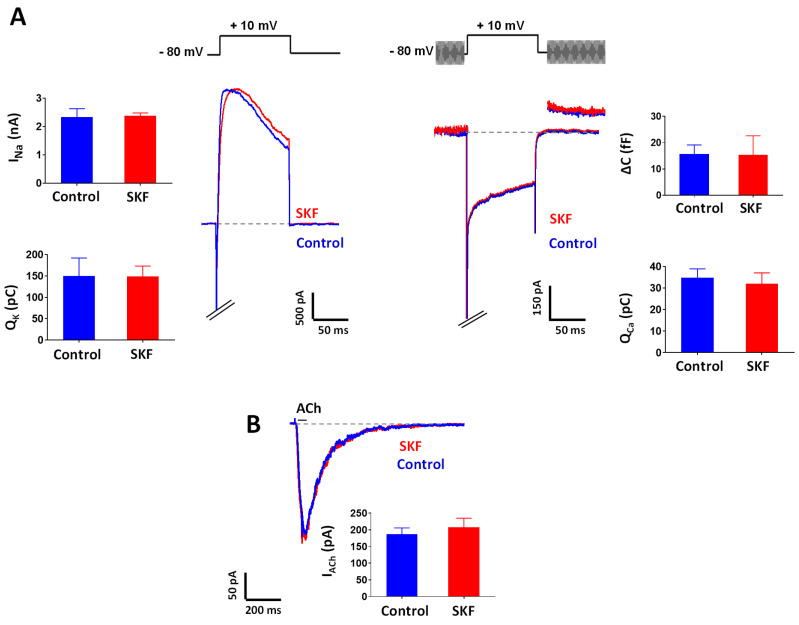
Effect of SKF29661 on ionic conductances and exocytosis in chromaffin cells from Control animals. (**A**) Effect of SKF29661 (300 μM, 2 min; SKF) on voltage-gated currents. *Left panel*. Representative voltage-gated Na^+^ and K^+^ currents evoked by a depolarization pulse (see voltage protocol on top of the recordings) before (Control) and after administration of SKF to a chromaffin cell in a tissue slice; bar graphs of the effect of SKF on peak amplitudes of Na^+^ currents (I_Na_) and charge of K^+^ currents (Q_K_) in chromaffin cells (*n* = 6 cells, two rats). *Right panel*. Representative voltage-gated Na^+^ and Ca^2+^ currents and the associated changes in membrane capacitance evoked by a depolarization pulse flanked by two sinusoid waves (±20 mV; 1 KHz; see voltage protocol on top of the recordings) before (Control) and after administration of SKF to a chromaffin cell in a tissue slice; bar graphs of the effect of SKF on the charge of Ca^2+^ currents (Q_Ca_) and the associated capacitance increments (∆C) (*n* = 6 cells, two rats). (**B**) Effect of SKF29661 (300 μM, 2 min; SKF) on acetylcholine (ACh)-evoked currents. Representative currents evoked by ACh (100 µM, 50 ms) before (Control) and after administration of SKF to a chromaffin cell in a tissue slice; bar graph of ACh-induced current amplitudes (I_ACh_) before (Control) and after SKF administration to chromaffin cells (*n* = 5 cells, two rats). V_h_ = −80 mV in A and B. Data are expressed as mean ± SEM. Statistical significance was assessed by the paired Student’s *t*-test. Non-significant differences (*p* > 0.05) are not shown.

To rule out a possible effect of SKF29661 on major ionic conductances involved in the exocytotic release of CAs from chromaffin cells, we performed patch–clamp recordings in tissue slices of the adrenal gland from Control, non-operated animals. At a concentration of 300 µM, the highest concentration reported to be used in functional studies in vitro [27], SKF29661 did not modify ionic currents through Na_v_, K_v_, and Ca_v_ channels of the membrane of chromaffin cells (Figure 5A). Moreover, SKF29661 did not affect exocytosis evoked by Ca^2+^ entry through Ca_v_ channels as assayed by membrane capacitance measurements (Figure 5A). Last, SKF29661 (300 µM) did not interfere with ACh-induced inward currents in chromaffin cells (Figure 5B), therefore, suggesting that this compound does not affect stimulus-secretion coupling in this neuroendocrine cell.

### 2.4. Effect of Adrenal Gland Denervation on Synaptic Activity of Chromaffin Cells and Mechanical Allodynia in CCI Animals 

**Figure 6:**
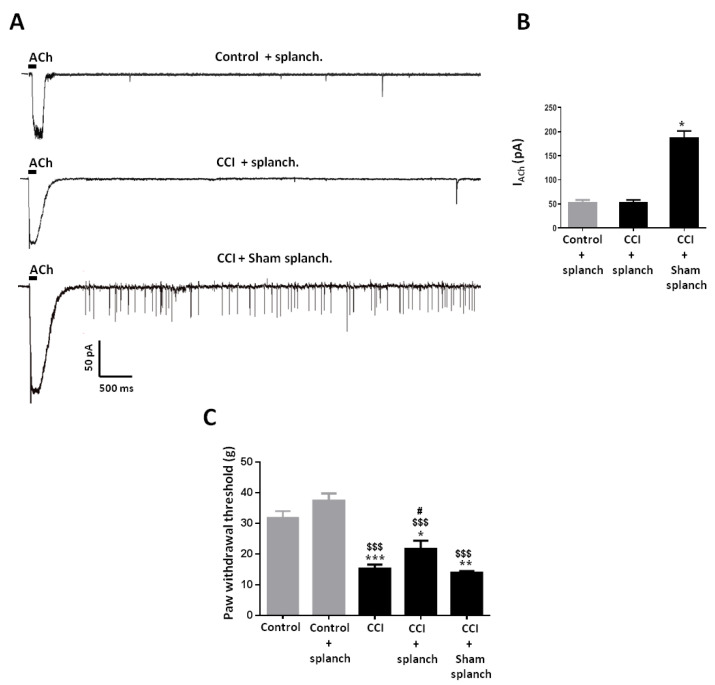
Effect of splanchnectomy on synaptic activity in chromaffin cells and mechanical allodynia from CCI animals. (**A**) Representative currents evoked by ACh (100 µM, 50 ms; see horizontal bar on top of the recordings) and spontaneous excitatory postsynaptic currents (sEPSCs) recorded in chromaffin cells from a Control animal subjected to splanchnectomy (Control + splanch.; upper record), a CCI animal subjected to splanchnectomy (CCI + splanch.; middle record), and a CCI animal subjected to sham splanchnectomy (CCI + Sham splanch., lower record). V_h_ = −80 mV. (**B**) Peak amplitudes of currents evoked by ACh (I_ACh_) in chromaffin cells from Control animals subjected to splanchnectomy (Control + splanch; *n* = 17 cells), CCI animals subjected to splanchnectomy (CCI + splanch; *n* = 15 cells), and CCI animals subjected to sham splanchnectomy (CCI + Sham splanch; *n* = 10 cells). *: *p* < 0.05 with regard to CCI + splanch (unpaired Student’s *t*-test). **C**. Paw withdrawal thresholds to mechanical stimulation in Control animals (Control; *n* = 9 rats), Control animals subjected to splanchnectomy (Control + splanch; *n* = 8 rats), CCI animals (CCI; *n* = 10 rats), CCI animals subjected to splanchnectomy (CCI + splanch; *n* = 6 rats), and CCI animals subjected to sham splanchnectomy (CCI + Sham splanch; *n* = 2 rats).*: *p* < 0.05; **: *p* < 0.01; ***: *p* < 0.001 with regard to Control. ^$$$^: *p* < 0.001 with regard to Control + Splanch. ^#^: *p* < 0.05 with regard to CCI (unpaired Student’s *t*-test).

The contribution of the adrenal medulla to behavioral manifestations of neuropathic animals was also assessed in animals subjected to bilateral splanchnectomy as a means to suppress neural stimulation of chromaffin cells. Upon denervation, chromaffin cells in tissue slices from the adrenal gland of CCI animals were practically devoid of synaptic activity as evidenced by the infrequent occurrence of sEPSCs, and showed reduced nicotinic currents evoked by exogenous application of ACh (100 μM; 50 ms) in comparison to cells from CCI animals undergoing sham surgery (Figure 6A). Interestingly, splanchnectomy also reduced the size of nicotinic currents in Control animals to a level similar to that of splanchnectomized CCI animals, suggesting that synaptic activity regulates the expression of nAChRs in chromaffin cells irrespectively of the stress condition of the animal (Figure 6B). Importantly, splanchnectomy partly reverted mechanical allodynia in CCI animals without affecting mechanical sensitivity in Control animals, which points to the role of adrenal medulla function in nociception only in neuropathic animals (Figure 6C).

## 3. Discussion

The two main observations communicated in this report are that: (i) inhibition of PNMT, the enzyme that converts NA into A, reverts mechanical allodynia in the CCI model of neuropathic pain in the rat; and (ii) adrenal medulla denervation (splanchnectomy) alleviates mechanical allodynia in the same experimental setting. Since both interventions affect adrenal medulla function by clearly distinct mechanisms, our results strongly support the involvement of this neuroendocrine tissue in the modulation of neuropathic pain secondary to injury of a peripheral nerve. Besides confirming that neuropathic pain acts as a stressor that mobilizes the sympathoadrenal arm of the SNS, our results imply that the stress response mediated by this arm influences pain behavior in a manner reminiscent of the notion of sympathetically maintained pain.

Previous results from our group have evidenced that the adrenal medulla of CCI animals undergoes profound functional and morphological remodeling leading to a stronger activation of chromaffin cells as manifested by a higher frequency of sEPSCs, increased firing of action potentials, and a more effective exocytosis evoked by voltage-gated Ca^2+^ entry. The present results also evidenced changes in the CAs content of the adrenal gland and blood, that translate into an increase in A/NA in adrenal (from 5.84 ± 0.28 in Control animals to 7.40 ± 0.48 in CCI ones) and blood (from 1.43 ± 0.32 in Control animals to 2.51 ± 0.81 in CCI ones) samples. It is pertinent now to recall that A and NA are synthesized, stored, and released from two separate chromaffin cell types, namely A- and NA-containing cells [32,33]. In the rat adrenal medulla, A-containing cells are about 85% of chromaffin cells, which precisely correlate with the A/NA of 5.84 ± 0.28 that we observed in the adrenal gland of Control animals. Importantly, different types of stressors may stimulate A- and NA-containing chromaffin cells differentially [34,35]. This is the case of hypoglycemia induced by 2-deoxi-D-glucose or insulin, which activates preferentially A-containing chromaffin cells in contrast to baroreceptor reflex activation or acute and chronic cold exposure, which predominantly stimulates NA-containing chromaffin cells [36,37,38]. Neuropathic pain evoked by CCI seems then to behave similarly to hypoglycemia and preferentially activate A-containing chromaffin cells.

We have investigated whether this change in A/NA might have a pathophysiological implication in CCI animals by assaying the effect of SKF29661, a highly selective and potent inhibitor of peripheral PNMT [27,31]. SKF29661 markedly reduced the A/NA in the adrenal gland and blood from Control (1.47 ± 0.22, and 1.34 ± 0.31) and CCI (2.16 ± 0.09, and 1.17 ± 0.16) animals, and, importantly, reverted mechanical allodynia only in the injured hind paw of CCI animals. As expected, SKF29661 increased the content of NA in the adrenal gland and blood samples from both Control and CCI animals and reduced the A content in the adrenal gland from Control animals and the blood from Control and CCI animals. Interestingly, SKF29661 increased the A content in the adrenal gland from CCI animals. This latter result could be explained by considering the competitive mechanism of action of SKF29661 [27] and the stimulatory effect of stress on CAs biosynthesis. It is well known that stress induces CAs biosynthesis in the adrenal gland in an attempt to prevent the depletion of CAs pools in the face of a sustained body demand [39]. This phenomenon, classically referred to as “stimulus-synthesis coupling”, involves several enzymes of the CAs biosynthetic pathway (tyrosine hydroxylase (TH), dopamine β-hydroxylase (DBH), and PNMT) whose activity is increased by an augmented secretion of adrenal corticosteroids and input from splanchnic nerve fibers [40,41,42,43]. In this context, one would expect to observe a marked increase in NA brought about by both an augmented production due to induction of TH and DBH and a reduced utilization by PNMT in the presence of SKF29661. In turn, the resultant accumulation of NA would compete with SKF29661, reducing its inhibitory effect on PNMT activity and, consequently, allowing a larger A synthesis.

The effects of SKF29661 appear to derive from selective inhibition of PNMT activity. Previous data indicated that it does not act on other CAs synthesizing (TH) and degrading (monoamine oxidase, catechol O-methyltransferase) enzymes, as well as it does not either activate or block α_1_, β_1,_ or β_2_ adrenoceptors [27,28]. Our results show that SKF29661 lacks an effect on motor coordination, is devoid of α_2_ adrenoceptor agonist activity, and left intact major ionic conductances in chromaffin cells while preserving exocytosis. Hence, all these evidences support the idea that SKF29661 acts by changing the relative content of the two (A and NA) adrenal CAs, without interfering with the release mechanism itself.

In agreement with the reversible nature of PNMT inhibition exerted by SKF29661, its effect on mechanical allodynia was also reversible, so that allodynia recovered between 24 h and 96 h after SKF29661 administration. The onset of the antiallodynic effect was fast (30 min) and lasted for a minimum of 24 h. Such a rapid action could possibly be explained because newly formed chromaffin vesicles storing CAs are paradoxically the first ones to be released by exocytosis upon stimulation [44,45]. Altogether, our behavioral data suggest that SKF29661 could act as a pain reliever instead of a disease-modifying agent, inhibiting spontaneous and evoked pain without affecting normal sensory mechanical sensitivity. SKF29661 administration also seems to be safe as denoted in a preclinical study in which in rats that were given oral doses of SKF29661 ranging from 150 to 600 mg/kg/day for 1 year, no significant toxic effects were noted; likewise, the low oral toxicity of this compound was also indicated by the fact that a lethal dose could not be obtained [28]. Altogether, these evidences point to SKF29661 and PNMT inhibition as a promising approach to reduce the unmet medical need represented by chronic neuropathic pain.

As a means to corroborate the involvement of the adrenal medulla in nocifensive behavior in CCI animals, we set out to denervate the adrenal medulla by sectioning the splanchnic nerves conveying preganglionic sympathetic input to chromaffin cells. This maneuver practically suppressed synaptic activity in chromaffin cells and reverted the increase in ACh-evoked currents observed in CCI animals. These two observations suggest that pain-evoked reflex stimulation of the adrenal medulla [46] was suppressed, thereby allowing the partial reversal of mechanical allodynia observed in splanchnectomized CCI animals. Chromaffin cells also secrete antinociceptive peptides (i.e., met-enkephalin, galanin) into the blood, which may partly counteract the algesic effect of CAs [2,47]. In fact, chromaffin cell transplants have been experimentally used as a cell therapy for neuropathic pain [48,49]. Since adrenal medulla denervation most likely impairs secretion of both CAs and antinociceptive peptides, it is not surprising that it alleviated only in part mechanical allodynia in CCI animals.

There have been previous reports that PNMT inhibition produces antinociception. So, DCMB (2,3-dichloro-α-methylbenzylamine), a PNMT inhibitor, potentiates analgesia produced by corticosterone and bee venom administration in the formalin model of inflammatory pain [50,51]. Interestingly, adrenalectomy mimicked the effect of PNMT inhibition, and systemic administration of A blocked the adrenalectomy-mediated enhancement of antinociception. Adrenaline is a well-known nociceptive agent that, upon acute and chronic administration, induces mechanical hyperalgesia. Its nociceptive effect appears to be mediated by β adrenoceptors located in primary nociceptive neurons since it is blocked by antagonists of β adrenoceptors [17,25].

In summary, our results strongly suggest the involvement of the adrenal medulla in mechanical allodynia in the CCI model of neuropathic pain. Inhibition of PNMT appears to be an effective and probably safe way to modulate adrenal medulla activity and, in turn, to alleviate pain secondary to the injury of a peripheral nerve. Importantly, this sort of treatment is devoid of antinociceptive activity, hence avoiding unwanted effects related to the loss of sensitivity to painful stimuli.

## 4. Materials and Methods

Adult male Sprague-Dawley rats (weighing 200–220 g/6–8 weeks old) were used in the experiments. Animals were housed in transparent cages with temperature-controlled at 23 °C in a 12-h light/dark cycle room; water and food were provided ad libitum. All experimental procedures were conducted according to the animal welfare guidelines of the European Community (European Directive 2010/63/UE) to minimize animal suffering and were approved by the Committee for Animal Experimentation of the Universidad Complutense de Madrid (approval date, 1 June 2011).

### 4.1. Chronic Constriction Injury of The Sciatic Nerve

The CCI model simulated the clinical condition of chronic nerve compression, such as the one that occurs in nerve entrapment neuropathy or spinal root irritation by a lumbar disk herniation. CCI produces a partial denervation of the sciatic nerve that affects myelinated A-fibers, while most unmyelinated C-fibers remain intact, hence allowing for the analysis of pain behaviors evoked by stimulation of the nerve’s target (the hind paw) [52,53]. CCI was performed according to Bennett and Xie (1988) [52]. Briefly, rats were anesthetized with intraperitoneal ketamine (100 mg/kg; Merial Labs, Spain) and medetomidine (100 µg/kg; Esteve Labs, Spain). Under sterile conditions, approximately 7 mm of the right nerve was freed proximal to the sciatic trifurcation, and four barely constricting ligatures (1 mm apart) using 4/0 chromic catgut were applied; in sham surgery, the nerve was exposed, but no ligatures were applied. The incision was closed in layers with silk thread 6/0. Animals were then allowed to recover from surgery for 7 days before being used in additional procedures, including adrenal gland denervation.

### 4.2. Adrenal Gland Denervation

Bilateral denervation of adrenal medulla was performed according to Miao et al. (2000) by sectioning the splanchnic nerves [54]. Following lateral incisions in the abdominal wall, the suprarenal ganglia and the nerves innervating the adrenal glands were exposed. The nerves connecting to the suprarenal ganglia were cut, and the ganglia gently removed so that major and minor splanchnic nerve outputs to the adrenal medulla were eliminated; in sham surgery, the nerves were exposed, but no sectioning was performed. CCI animals underwent denervation or sham-denervation at day 7–8 post-CCI surgery. Animals were anesthetized as described for CCI surgery and allowed to recover from surgery for 5 days before being used in additional procedures.

### 4.3. Behavioural Testing

#### 4.3.1. Mechanical Allodynia

Rats were habituated to the experimental setting for at least 30 min before testing. All tests were conducted between 09:00 and 12:00. Mechanical allodynia was evaluated with a dynamic plantar aesthesiometer (Ugo Basile, Gemonio, Italy) by means of a 0.5 mm filament exerting increasing force (up to 50 g over 20 s) onto the plantar surface of the hind paw until the animal withdrew its paw, the actual force at that time was automatically registered (paw withdrawal threshold; PWT). Hypersensitivity was defined as at least a 25% decrease in PWT compared with values before CCI surgery. PWT measurements were repeated 3 times at 5 min intervals, and the mean value was reported. Rats not exhibiting mechanical hypersensitivity were discarded.

PWT determination was carried out before surgery (mean of 3 measurements on alternate days the week preceding surgery, collectively designated as day −1) and on post-surgery days 7–21, when abnormal pain behavior was at a stable maximum. Likewise, responses to mechanical stimulation were assessed before and 30 min after intraperitoneal (250 µL; I.P.) drug injection with a Hamilton^®^ syringe with a 30G gauge needle. Control responses were obtained with vehicle (saline solution), and drugs used were atipamezole (Tocris, Bristol, UK) and 1,2,3,4-tetrahydroisoquinoline-7-sulfonamide (SKF29661), a kind gift of Glaxo Smith Kline, Philadelphia, USA.

#### 4.3.2. Motor Coordination

Motor coordination was assessed with a RotaRod apparatus (Ugo Basile, Gemonio, Italy). Rats were trained in the experimental procedure for at least two days. Motor coordination was evaluated through the time the animal spent on a roller rotating at a continuous speed (16 rpm). The cut-off time was 90 s divided into two intervals of 45 s [55]. Only non-operated animals were evaluated. After obtaining baseline values, the animals were treated with SKF29661 or vehicle.

### 4.4. Adrenal Gland Preparation

Animals were sacrificed by cervical dislocation followed by decapitation, and the two adrenal glands were extracted and used in either functional experiments or for CA determination (see below). Acute tissue slices of the adrenal gland were prepared as previously described [6]. After removal, the glands were sagitally sectioned with a vibratome (Integraslice 7550 MM, Campden Instruments, Loughborough, UK) to obtain 300 μm-thick slices (6–8 slices per gland). Slices were then transferred to a storage chamber containing Ringer’s saline of the following composition (in mM): 125 NaCl, 2.5 KCl, 2 CaCl_2_, 1 MgCl_2_, 1.25 NaH_2_PO_4_, 26 NaHCO_3_, and 12 glucose (pH 7.4, adjusted with HCl; ≈300 mOsm) continuously bubbled with carbogen (95% O_2_/5% CO_2_) at room temperature for a maximum of 6 h.

### 4.5. Electrophysiological Recordings

Slices were fixed with a nylon grid to the bottom of a chamber attached to the stage of an upright microscope (Olympus BX51W1, Barcelona, Spain) and continuously superfused with Ringer’s solution at a rate of approximately 1 mL × min^−1^. Cells were viewed under a 63× water immersion objective and a DL-604 OEM camera (Andor Technology, South Windsor, CT, USA). All electrophysiological recordings were performed in the perforated-patch variant of the whole-cell configuration of the patch–clamp technique with an EPC10/2 amplifier using PatchMaster software (HEKA Electronic, Lambrecht, Germany) [56]. Patch pipettes were made from borosilicate glass and fire-polished to a resistance of 5.5–8.5 MΩ when filled with an internal solution. The standard internal recording solution had the following composition (mM): 145 KCl, 2 MgCl_2_, 0.3 EGTA, 0.3 GTP.Li_3_, 2 ATP.Na_2_, 10 HEPES 10 (pH 7.2 adjusted with KOH ≈280 mOsm). The internal solution used to isolate voltage-gated Ca^2+^ currents and the associated membrane capacitance changes were (mM): 145 CsCl, 8 NaCl, 1 MgCl_2_, 2 ATP.Na_2_, 0.3 GTP.Li_3_, 0.3 EGTA, 10 HEPES (pH 7.2 adjusted with CsOH; ≈280 mOsm). Membrane currents were filtered at 1 (sEPSCs and ligand-activated currents) or 3 (voltage-activated currents) kHz and sampled at 10 kHz. Perforated-patch recordings were done with pipettes immersed for a few seconds into a plain internal solution and then back-filled with the same internal solution containing amphotericin B (400 µg/mL, Sigma–Aldrich, Madrid, Spain). The quantity of charge, Q, carried by voltage-activated currents was calculated as the time integral of the inward (Ca^2+^) or outward (K^+^) current evoked by a voltage pulse to +10 mV (100 ms). Given the presence of an early inward Na^+^ current, the limits for the current integration were fixed 3–5 ms after the beginning of the pulse, once 80% of the Na^+^ current had decayed, and excluded the tail currents. Exocytosis was estimated by the membrane capacitance increment (ΔC) evoked by the same depolarizing step according to the Lindau–Neher technique implemented as the “Sine + DC” feature of the PatchMaster software [53]. The *p*/n method with *p*/4 pulses was used in all protocols with rapid changes in the potential for the automatic subtraction of capacitive and leak currents. Drugs were applied by either bath perfusion (SKF29661 300 µM; 2–10 min) or by means of a pneumatic ejection system (PDES-02DX, NPI Electronic GmbH, Germany) from a puffer pipette with an opening of around 3–5 μm placed near (5–10 μm) the cell under study (ACh; 100 µM, 50 ms). Experiments were carried out at room temperature (22–25 °C).

### 4.6. Determination of Catecholamines 

#### 4.6.1. Adrenal Gland Samples

Adrenal glands were placed in a cold Ca^2+^- and Mg^2+^-free Hank’s solution (Sigma–Aldrich, Spain) and transferred to 500 μL of 0.1 N perchloric acid before being sonicated (Sonics material vibracell, Dambury, CT, USA) and, subsequently, centrifuged at 3000 rpm for 10 min at 4 °C (tabletop centrifuge, Hettich, Germany). The supernatant was collected, centrifuged at 3000 rpm for 2 min at 4 °C (Eppendorf, Germany), and analyzed by liquid chromatography-tandem mass spectrometry (LC-MS/MS). A system consisting of a 1200 Series Liquid Chromatograph coupled to a 6410B Triple Quadrupole Mass Spectrometer (LC-MS; Agilent Technologies, Madrid, Spain) was used [57], and CAs were determined using a positive mode electrospray ionization; likewise, the pellet was analyzed for protein content.

#### 4.6.2. Blood Samples

Trunk blood was collected in plastic tubes immediately following decapitation. Blood was kept at 4 °C and centrifuged at 2000 rpm for 10 min. Then, the supernatant was aspirated in aliquots of 250 μL and transferred into Eppendorf´s tubes that were kept frozen at −80 °C. After protein precipitation and double centrifugation at 3000 rpm for 5 min at 4 °C, CAs were determined using the LC-MS/MS technique.

### 4.7. Statistics 

Data are given as the mean ± standard error of the mean (SEM) of the corresponding number of cells and/or animals evaluated. In behavioral experiments, differences between groups were assessed by one or two-way analysis of variance (ANOVA) for repeated measurements, with Tukey’s post-test comparisons. Paired or unpaired Student’s *t*-tests were used for data comparisons from electrophysiological and biochemical experiments. GraphPad Prism 5 (GraphPad Software, La Jolla California, USA) was employed for these analyses. Differences with *p* < 0.05 (*) were considered significant; ** indicates *p* < 0.01, and *** indicates *p* < 0.001.

## Abbreviations

A	Adrenaline
A/NA	A to NA ratio
ACh	Acetylcholine
ANOVA	Analysis of variance
CA	Catecholamine
Ca_v_	Voltage-gated calcium channels
CCI	Chronic constriction injury
ΔC	Capacitance increment
DCMB	2,3-dichloro-α-methylbenzylamine
I.P.	Intraperitoneal
K_v_	Voltage-gated potassium channels
LC-MS	Liquid Chromatograph Mass Spectrometer
nAChR	Nicotinic acetylcholine receptor
NA	Noradrenaline
Na_v_	Voltage-gated sodium channels
PNMT	Phenylethanolamine N-methyltransferase
PWT	Paw withdrawal threshold
sEPSC	Spontaneous excitatory synaptic currents
SKF29661	1,2,3,4-tetrahydroisoquinoline-7-sulfonamide
SNS	Sympathetic nervous system
TH	Tyrosine hydroxylase
V_h_	Holding potential
